# Comparing the Long-Term Cardiovascular Outcomes of Lumbo-Peritoneal, Ventriculo-Peritoneal, or Non-Shunting Treatment after Idiopathic Normal Pressure Hydrocephalus: A Nationwide Retrospective Cohort Study

**DOI:** 10.7150/ijms.92032

**Published:** 2024-01-27

**Authors:** Cheng-Di Chiu, Po-Fan Chiu, Chih-Ying Wu, Hei-Tung Yip, You-Pen Chiu, Hui-Ru Ji, Kai-Cheng Hsu, Fuu-Jen Tsai, Robert Chen-Hao Chang

**Affiliations:** 1 Spine Center, China Medical University Hospital, Taichung, Taiwan.; 2 Department of Neurosurgery, China Medical University Hospital, Taichung, Taiwan.; 3 School of Medicine, College of Medicine, China Medical University, Taichung, Taiwan.; 4 Graduate Institute of Biomedical Science, China Medical University, Taichung, Taiwan.; 5 Graduate Institute of Medical Sciences, National Defense Medical Center, Taipei, Taiwan.; 6 Department of Neurosurgery, China Medical University Hsinchu Hospital, Hsinchu, Taiwan.; 7 Graduate Institute of Integrated Medicine, China Medical University, Taichung, Taiwan.; 8 School of Chinese Medicine, College of Chinese Medicine, China Medical University, Taichung, Taiwan.; 9 Department of Medical Research, China Medical University Hospital, China Medical University, Taichung, Taiwan.; 10 Division of Medical Genetics, China Medical University Children's Hospital, Taichung, Taiwan.; 11 Department of Biotechnology and Bioinformatics, Asia University, Taichung, Taiwan.; 12 Artificial Intelligence Center for Medical Diagnosis, China Medical University, Taichung, Taiwan.; 13 Department 0f Neurology, China Medical University Hospital, Taichung, Taiwan.; 14 Management Office for Health Data, China Medical University Hospital, Taichung, Taiwan.; 15 Department of Electrical Engineering, National Chung Hsing University, Taichung 40227, Taiwan.; 16 Department of Electrical Engineering, National Chung Hsing University, Taichung, Taiwan.

**Keywords:** idiopathic normal pressure hydrocephalus, lumbo-peritoneal shunt, major adverse cardiovascular events, ventriculo-peritoneal shunt

## Abstract

**Purpose:** With advances in medical technology, the average lifespan has increased, leading to a growing significance of idiopathic normal pressure hydrocephalus (iNPH), particularly in the elderly population. Most patients with iNPH have been treated either with ventriculo-peritoneal shunts (VPS) or conservative measures. However, lumbo-peritoneal shunts (LPS) have emerged as an alternative treatment option for iNPH in recent decades, extensive research still lacks comparing outcomes with LPS to those with VPS or non-surgical treatment. The aim of the resent study is to disclose the long-term therapeutic outcomes of LPS, VPS, and non-shunting in patients with iNPH.

**Methods:** We used the National Health Insurance Research Database in Taiwan to assess the long-term outcomes of these treatment options. We enrolled 5,537 iNPH patients who received shunting surgery, of which 5,254 were VPS and 283 were LPS. To compare the difference between each group, matching was conducted by propensity score matching using a 1:1 ratio based on LPS patients. Primary outcomes included death and major adverse cardiovascular events (MACEs)

**Results:** Our findings show that VPS resulted in significantly more MACEs than non-surgical treatment (Odds ratio: 1.83, 95% confidence interval: 1.16-2.90). In addition, both VPS and LPS groups had significantly lower overall mortality rates than non-shunting group. Moreover, LPS had lower overall mortality but similar MACEs rates to VPS.

**Conclusions:** Based on these findings, we propose that the LPS is preferable to the VPS, and surgical treatment should be considered the primary choice over conservative treatment unless contraindications are present.

## Introduction

Idiopathic normal pressure hydrocephalus (iNPH) was first described by Salomon Hakim Doe and Raymond Delesy Adams in 1965. It is one type of communicating hydrocephalus characterized by enlarged brain ventricles with normal cerebrospinal fluid (CSF) pressure 1-4. The cause of most iNPH cases is unknown, but some evidence indicates that it might be related to increased arterial pulse pressure 5, 6. Patients with iNPH typically have clinical triads, also known as the Hakim-Adams triads, which includes gait imbalance, dementia, and urinary incontinence 7. While previous intracerebral hemorrhage, traumatic brain injury, and intracranial infection are recognized as risk factors for iNPH, it most commonly develops without an identifiable cause, particularly in elderly patients 8, 9. The prevalence of iNPH in the elderly population has been reported to be 1.4%-2.0%, and it tends to increase with age 10, 11. Given the increasing proportion of elderly individuals in society, accurate diagnosis without delay and appropriate treatment of iNPH have become increasingly important in recent years 12.

Currently, there remains no effective conservative treatment available for patients with iNPH. While acetazolamide can temporarily reduce interstitial brain water by increasing cerebral blood flow, it is not a long-term solution 3. The traditional and effective surgical treatment for iNPH is CSF diversion, which involves placing ventriculo-peritoneal (VPS) or ventriculo-atrial (VAS) shunts 8. CSF shunt placement success rates in iNPH range from 70% to 96%, indicating favorable outcomes for many patients 13, 14. However, it is important to acknowledge the potential complications associated with these intracranial procedures, including cardiovascular events, infections, shunt obstruction, revisions, and epilepsy, which cannot be overlooked 8, 15.

The lumbo-peritoneal shunt (LPS) is an alternative treatment option for iNPH that was first used as early as 1949 16, 17. Only around 5% of patients with iNPH undergoing shunting in Taiwan received an LPS. Notably, only one LPS device has entered clinical use since 2011 (PS Medical Strata NSC Lumbo-peritoneal Valve and Shunt System; Medtronic, Cremona Drive, Goleta, CA, USA), which was approved by the US Food and Drug Administration in 2009 based on their individual safety and efficacy data. The LPS offers a more minimally invasive and distinct approach than ventricular puncture with VPS or VAS, and several studies have reported its safety and effectiveness in alleviating iNPH symptoms 8, 18, 19. However, there is a limited number of real-world studies directly comparing surgical outcomes between VPS and LPS in iNPH treatment 2, 8, 15, 20, 21. Therefore, this cohort study aims to evaluate major cardiovascular events (MACEs) and mortality in patients with iNPH treated with either VPS or LPS.

## Materials and Methods

### Data source

The National Health Insurance Research Database (NHIRD) is among Taiwan's precious medical data sources. It contains the medical record of almost all residents of Taiwan since 1995 when a single-payer National Health Insurance program was launched. Their time of disease onset, medicine usage, and operation date are available for investigation. The database uses diagnostic codes from the International Classification of Diseases, Ninth and Tenth Revision, Clinical Modification (ICD-9/10-CM). This study was approved by the Institutional Review Board of China Medical University Hospital Research Ethics Committee (approval number: CMUH109-REC2-031 [CR-2]).

### Study population

**Figure 1:**
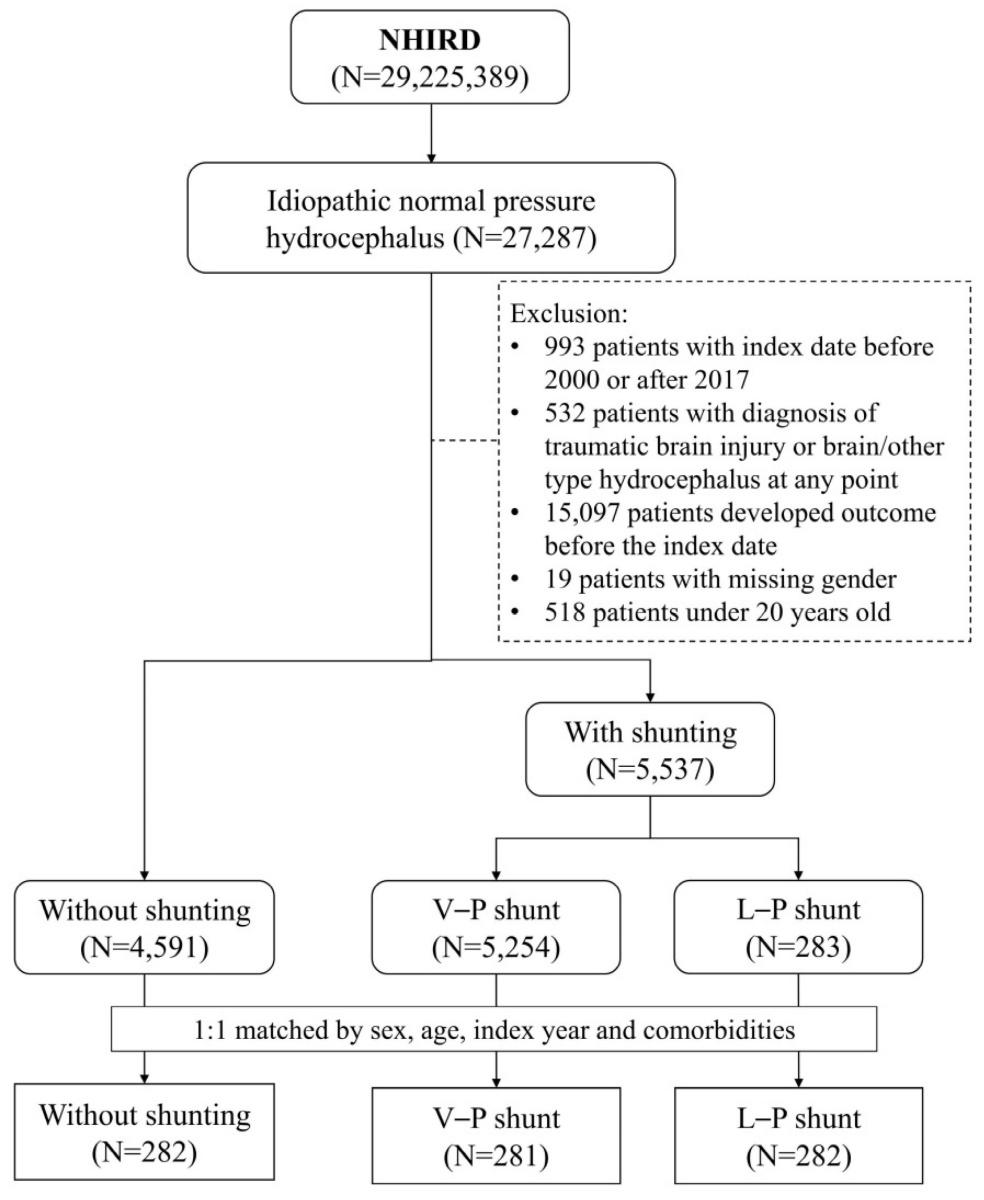
Flow chart.

This study enrolled patients diagnosed with iNPH (ICD-9-CM code: 331.3; ICD-10-CM code: G91.2) between 2000 and 2017. They were divided into three groups: (1) the control group without shunting, (2) VPS, and (3) LPS. The index date was the date of the shunt operation for patients receiving a shunt and a random date after the iNPH diagnosis for the control group. We excluded patients aged <20 years, with traumatic brain injury or brain another type of hydrocephalus, or hospitalized for major adverse cardiovascular events (MACEs) before the index date. We selected the control and VPS patients at a 1:1 ratio according to LPS patients (Figure 1). Three groups matching was conducted by a logistic regression model and propensity score matching. Using a greedy matching algorithm adjusted for factors such as age, sex, index year, and comorbidities including coronary artery disease (CAD), hypertension, hyperlipidemia, chronic obstructive pulmonary disease (COPD), chronic kidney disease (CKD), diabetes mellitus (DM), Alzheimer's disease (AD), and dementia.

### Main outcome and comorbidities

The primary outcome was a MACE, including ischemic stroke (ICD-9-CM code: 433-436; ICD-10-CM codes: I63, I65, I66, I67.8, G45, and G46), hemorrhagic stroke (ICD-9-CM code: 430-432; ICD-10-CM codes: I60, I61, and I62), acute myocardial infarction (AMI; ICD-9-CM code: 410; ICD-10-CM code: I22), congestive heart failure (CHF; ICD-9-CM code: 428; ICD-10-CM codes: I50.0-I50.4 and I59). Mortality was also an outcome. Related comorbidities were CAD (ICD-9-CM code: 411-414; ICD-10-CM codes: I20, I24, and I25), hypertension (ICD-9-CM code: 401-405; ICD-10-CM codes: I10, I11, I12, I13, I15, and N262), atrial fibrillation (ICD-9-CM code: 427.31; ICD-10-CM code: I48.0), hyperlipidemia (ICD-9-CM code: 272.0-272.4; ICD-10-CM code: E780-E785), COPD (ICD-9-CM codes: 491, 492, and 496; ICD-10-CM codes: J41, J42, J43, and J44), CKD (ICD-9-CM code: 580-588; ICD-10-CM code: N18), DM (ICD-9-CM code: 250; ICD-10-CM code: E11), AD (ICD-9-CM code: 331.0; ICD-10-CM codes: G300, G301, G308, and G309), and dementia (ICD-9-CM code: 290; ICD-10-CM codes: F01, F03, F05, and F28).

### Statistical analysis

The distributions of sex, age, and comorbidities are shown as count and percentage; age is also expressed as the mean and standard deviation. The two groups were compared using the chi-square test for categorical variables and Student's *t*-test for continuous variables. The incidence rate was calculated by dividing the number of events by the person-years. The hazard ratio (HR) was obtained from a Cox proportional hazards model; the 95% confidence interval (CI) was also calculated. The cumulative incidence curve obtained using the Kaplan-Meier method was plotted and compared using the log-rank test. All analyses were conducted using SAS software (version 9.4; SAS Institute Inc., Cary, NC, USA). All results with a *p*-value <0.05 were considered statistically significant.

## Results

This cohort study enrolled 281 patients with iNPH with a VPS and 282 with an LPS. All baseline characteristics of the three groups were similar (Table 1). Over half of the subjects were male. Most subjects were aged >50 years, and their mean age was around 67 years. Over 70% of subjects had hypertension, about 50% had a history of hyperlipidemia, and 45% had DM. Other common comorbidities were 35% had CAD, 30% had dementia, 25% had COPD and 25% had CKD.

**Figure 2:**
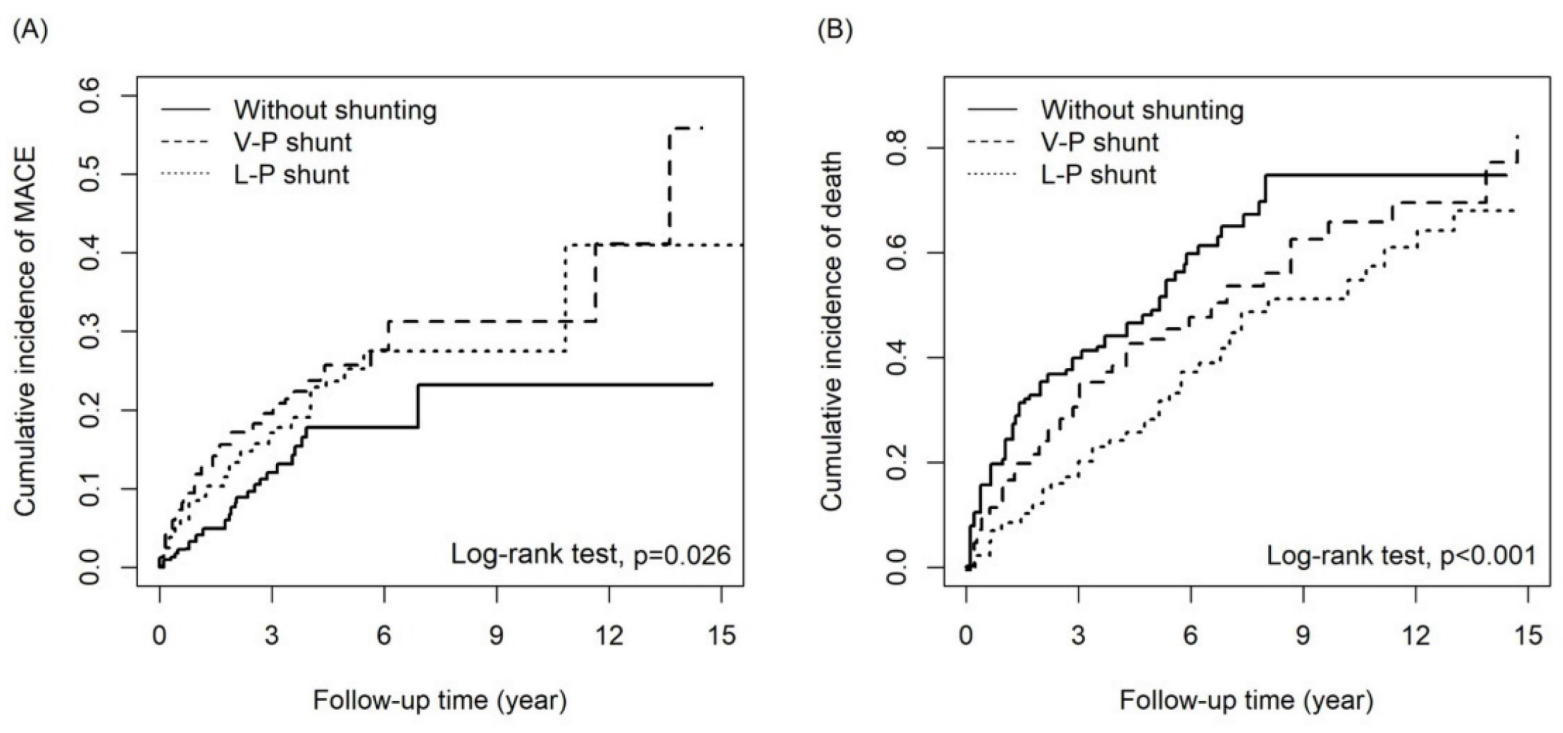
The cumulative incidence of (A)MACE and (B) death among three groups.

Patients with a VPS had a higher risk of MACEs than patients without shunting (adjusted HR [aHR] = 1.83, 95% CI = 1.16-2.90; p=0.010), notably hemorrhagic stroke (aHR = 3.20, 95% CI = 1.37-7.47; p = 0.007). Patients with a VPS had a lower mortality risk than patients without shunting (aHR = 0.74, 95% CI = 0.57-0.95; p=0.02). MACE risk did not differ significantly between patients with an LPS and those without shunting (aHR = 1.41, 95% CI = 0.89-2.24; p = 0.147). However, the difference in the risk of hemorrhagic stroke between patients with an LPS and those without shunting was marginally significant (aHR = 2.42, 95% CI = 1.00-5.86; p = 0.05). Notably, patients with an LPS had a 54% lower mortality risk than those without shunting (aHR = 0.45, 95% CI = 0.34-0.59. p<0.001). The HRs of other outcomes are listed in Table 2. Compared to patients with a VPS, patients with an LPS had a lower risk of CHF (aHR = 0.48, 95% CI = 0.25-0.96; p = 0.037) and mortality (aHR = 0.62, 95% CI = 0.47-0.81; p<0.001, Table 3). The cumulative incidence of MACEs and death in the three groups are presented in Figures 2a and 2b, respectively; the curves differed significantly between groups (cumulative incidence of MACEs are VPS>LPS>without shunting, with Log Rank test P=0.026; cumulative incidences of death are without shunting>VPS>LPS, with Log Rank test p<0.001).

Table 4 shows the results of stratification by follow-up time. In the first year, patients with a VPS had a 4.21-fold higher MACE risk (95%CI = 1.96-9.06) and 48% lower mortality risk (95% CI = 0.35-0.76) than without shunting group. In follow-up years 1-3, patients with a VPS had a higher mortality risk (aHR = 1.75, 95% CI = 1.08-2.83) than without shunting group. In the first year, patients with an LPS had a 70% lower mortality risk (aHR = 0.30, 95% CI = 0.19-0.47) than without shunting group. Table 5 shows the mean follow-up time of different outcomes in the control, VPS, and LPS groups.

## Discussion

Few studies on iNPH, a neurological disorder commonly treated with VPS or LPS, have compared real-world, long-term data on MACEs and mortality between these treatment options. In our study, we aimed to compare the rates of MACE, including ischemic stroke, hemorrhagic stroke, AMI, and CHF, and mortality among patients with iNPH who received a VPS, an LPS, or non-surgical management. There were 27,287 patients diagnostically coded with iNPH in the NHIRD. We excluded patients coded with traumatic brain injury or other types of hydrocephalus. This study included 282 patients who received an LPS, matched with 281 patients who received a VPS and 282 who received non-surgical management, adjusted for factors such as age, sex, index year, and comorbidities including CAD, hypertension, hyperlipidemia, COPD, CKD, DM, AD, and dementia.

Our results showed a significantly higher MACE rate in the VPS group than in the non-surgical management group (*P* < 0.01; Table 2). Hemorrhagic stroke was identified as the most causal factor of MACEs in the VPS group (*P* < 0.01), with 3.2 times the aHR than the non-surgical management group. This increased risk of hemorrhagic stroke could result from the ventricular puncture required for VPS or the changes in intracranial pressure after VPS placement 22-25. Furthermore, our study showed that most MACEs were more likely to occur within the first year of follow-up in the VPS group (*P* < 0.001; Table 4), consistent with clinical observations that surgical-related hemorrhagic complications mostly occur within the first year 22, 26. However, the overall mortality rate was lower in the VPS group than in the non-surgical group (*P* < 0.05). This lower mortality may be attributed to improved iNPH symptoms and functional status after shunting, which could decrease the risk of fatal falls or related disability. In addition, the lower mortality was particularly prominent within the first year after the operation (*P* < 0.001). These results showed that besides surgical complications (which are not usually life-threatening), treating patients with iNPH using a VPS was beneficial, including functional improvements as soon as several weeks after the operation 11, 27. However, mortality was higher in the VPS group than in the non-surgical group during the first to third years following VPS placement. This higher mortality can be attributed to life-threatening complications, including late infection, bowel perforation, delayed intracerebral or subdural hemorrhage, and delayed diagnosis of shunt malfunction, which are more frequently observed months to years after VPS placement. Moreover, the benefits of shunting in patients with iNPH decrease over time 25, 28.

While the MACE rate was slightly higher for the LPS group than the non-surgical management group, the difference was nonsignificant (Table 2). The incidence of hemorrhagic stroke was only slightly higher in the LPS group than in the non-surgical management group (*P* < 0.05). In addition, the HR for hemorrhagic stroke was lower in the LPS group than in the VPS group, although the difference was nonsignificant. The LPS procedure might be simpler because it is more minimally invasive and does not require ventricular puncture, although it still has the risk of over-drainage 15, 29, 30. In addition, mortality was lower in the LPS group than in the VPS group (*P* < 0.05), which can be attributed to improved iNPH symptoms, safer procedures, fewer adverse effects, and overall lower HRs for MACEs. This lower mortality was particularly notable within the first year of follow-up (*P* < 0.001), consistent with clinical observations 11, 27.

We also found no significant difference in MACEs between the VPS and LPS groups. However, the incidence of CHF was higher in the VPS group (*P* < 0.05; Table 3). The exact reason for the increased risk of CHF in the VPS group is unclear. However, Caplan et al. proposed a medical hypothesis for cardiac encephalopathy that connects CHF and iNPH 31. Nevertheless, it is unclear why this phenomenon was not observed in the LPS group. One possible explanation relates to the healthcare system in Taiwan, where the cost of an LPS device is not covered by insurance and is self-paid, making the LPS procedure more expensive than the insurance-covered VPS. Consequently, most patients who opt for an LPS may have better family support or higher economic status and can access more medical aids 20.

Our findings show that both VPS and LPS decrease the mortality of patients with iNPH compared to patients without shunting. Furthermore, they indicate that LPS is significantly more effective than VPS in reducing mortality (Tables 2, 3, and 4). The significantly higher incidences of CHF and hemorrhagic stroke in the VPS group may have resulted in higher mortality (*P* < 0.001) compared to the LPS group (Table 4). While most of the significant MACEs occurred within one year in both the VPS and LPS groups, their one-year mortality rates were still significantly lower than that of the non-surgical group (Table 4). These findings suggest that a more aggressive therapeutic approach, rather than conservative treatment alone, can be considered for iNPH. Moreover, LPS is preferable to VPS due to its lower incidence of hemorrhagic stroke and significantly lower rates of CHF and mortality.

While this study provides evidence of lower mortality and cumulative incidence of death in the LPS group compared to the VPS and non-surgery groups, it had several limitations (Figure 2B). Firstly, this retrospective cohort study could not assess iNPH severity and objective measures such as the Glasgow Coma Scale, laboratory data, and imaging studies, which could have influenced the outcomes. Secondly, the accuracy of the iNPH diagnosis was difficult to verify, and the completeness of the data may vary across different hospitals and clinics, potentially leading to errors or missing information. However, it should be noted that the NHIRD covers a highly representative sample of Taiwan's general population, and the diagnoses undergo scrutiny by medical reimbursement specialists and peer review, enhancing their reliability. Thirdly, information on medications that may have impacted iNPH and MACE outcomes was inaccessible due to limitations in the data sources. Fourthly, the study lacked information on influential risk factors such as physical activity, dietary habits, education, and social engagement, which could have affected the interpretation of the results. Fifthly, the evaluation of outcomes was limited to MACEs and mortality, with no assessment of other aspects of patient outcomes, such as quality of life or functional performance.

## Conclusion

Our study indicates that both VPS and LPS are effective treatments for iNPH and significantly reduce mortality. However, it should be noted that VPS may carry a higher risk of MACEs and CHF, which could contribute to higher mortality compared to LPS. Nevertheless, LPS may be a viable alternative with significantly lower mortality than VPS. However, caution should be exercised concerning the risk of hemorrhagic stroke associated with LPS. It is crucial to conduct further research to validate our findings and explore potential strategies for mitigating the risks related to shunt placement for patients with iNPH.

## Figures and Tables

**Table 1:** Baseline characteristics of the study cohort.

	Idiopathic normal pressure hydrocephalus patients								
	Without shunting			VPS		Compared to patients without shunting	LPS		Compared to patients without shunting
	N=282			N=281			N=282		
Variables	n	%		n	%	p-value	n	%	p-value
Sex						0.189			0.349
Female	114	40.43		129	45.91		125	44.33	
Male	168	59.57		152	54.09		157	55.67	
Age						0.644			0.816
20-30	17	6.03		22	7.83		18	6.38	
30-40	18	6.38		13	4.63		13	4.61	
40-50	10	3.55		8	2.85		9	3.19	
>50	237	84.04		238	84.70		242	85.82	
Mean, (SD) ^#^	66.6	17.57		67.15	17.76	0.716	67.53	16.99	0.525
Comorbidities									
CAD	92	32.62		110	39.15	0.107	111	39.36	0.096
Hypertension	202	71.63		211	75.09	0.353	207	73.40	0.637
Hyperlipidemia	146	51.77		136	48.40	0.423	149	52.84	0.800
COPD	69	24.47		67	23.84	0.863	74	26.24	0.628
CKD	68	24.11		65	23.13	0.784	66	23.40	0.843
DM	129	45.74		127	45.20	0.896	128	45.39	0.933
AD	13	4.61		15	5.34	0.691	16	5.67	0.567
Dementia	80	28.37		79	28.11	0.946	86	30.50	0.579

SD: standard deviation; LPS: lumboperitoneal shunt; VPS: ventriculoperitoneal shunt; CAD: coronary artery disease; COPD: chronic obstructive pulmonary disease; CKD: chronic kidney disease; DM: diabetes mellitus; AD: Alzheimer's disease; #: Student's t-test.

**Table 2:** MACE and mortality risk among the sampled patients with or without shunting.

	Without shunting						VPS					cHR	(95% CI)	p-value	aHR ^ꝉ^	(95% CI)	p-value
Outcome	N		PY		IR		N		PY		IR						
MACE	28		715		39.2		61		868		70.3	1.84	(1.18, 2.89)**	0.008	1.83	(1.16, 2.90)**	0.010
Ischemic stroke	13		738		17.6		25		941		26.6	1.60	(0.82, 3.13)	0.170	1.48	(0.74, 2.97)	0.270
Hemorrhagic stroke	7		751		9.32		25		940		26.6	3.01	(1.30, 6.97)*	0.010	3.20	(1.37, 7.47)**	0.007
ICH	<=3						7		995		7.04	1.75	(0.44, 6.87)	0.425	1.67	(0.42, 6.70)	0.471
SAH	<=3						<=3					0.52	(0.09, 3.12)	0.475	0.77	(0.12, 5.08)	0.785
AMI	4		758		5.28		<=3					0.60	(0.13, 2.66)	0.497	0.44	(0.07, 2.94)	0.396
CHF	12		742		16.2		22		976		22.5	1.41	(0.69, 2.85)	0.346	1.32	(0.63, 2.77)	0.466
Mortality	132		759		174.0		123		1009		121.9	0.74	(0.58, 0.95)*	0.018	0.74	(0.57, 0.95)*	0.020
MACE	28	715		39.2			57	1004		56.8		1.49	(0.95, 2.36)	0.084	1.41	(0.89, 2.24)	0.147
Ischemic stroke	13	738		17.6			33	1052		31.4		1.77	(0.93, 3.39)	0.082	1.68	(0.87, 3.25)	0.124
Hemorrhagic stroke	7	751		9.32			21	1112		18.9		2.14	(0.91, 5.05)	0.082	2.42	(1.00, 5.86)*	0.050
ICH	<=3						7	1145		6.1		1.61	(0.41, 6.27)	0.491	1.70	(0.41, 7.08)	0.464
SAH	<=3						4	1154		3.5		0.90	(0.20, 4.03)	0.888	1.01	(0.19, 5.24)	0.992
AMI	4	758		5.28			<=3					0.36	(0.07, 1.98)	0.240	0.55	(0.08, 3.81)	0.542
CHF	12	742		16.2			16	1144		14.0		0.90	(0.42, 1.92)	0.789	0.74	(0.34, 1.60)	0.442
Mortality	132	759		174			89	1168		76.2		0.48	(0.37, 0.63)***	<0.001	0.45	(0.34, 0.59)***	<0.001
																	

PY: person-year; IR: incidence rate per 100 person-years; cHR: crude hazard ratio; aHR: adjusted hazard ratio; CI: confidence interval; MACE: major adverse cardiovascular event; ICH: intracerebral hemorrhage; SAH: subarachnoid hemorrhage; CHF: congestive heart failure; VPS: ventriculoperitoneal shunt; LPS: lumboperitoneal shunt; ^ꝉ^: adjusted by sex, age, and all comorbidities; *: *p* < 0.05; ***: *p* < 0.001.

**Table 3:** MACE and mortality risk among the sampled patients with different shunting approaches.

	VPS				LPS			cHR	(95% CI)	p-value	aHR ^ꝉ^	(95% CI)	p-value
Outcome	N	PY	IR		N	PY	IR						
MACE	61	868	70.3		57	1004	56.8	0.83	(0.58, 1.20)	0.327	0.80	(0.55, 1.16)	0.239
Ischemic stroke	25	941	26.6		33	1052	31.4	1.20	(0.71, 2.01)	0.501	1.20	(0.70, 2.03)	0.509
Hemorrhagic stroke	25	940	26.6		21	1112	18.9	0.75	(0.42, 1.35)	0.341	0.75	(0.42, 1.35)	0.340
ICH	7	995	7.04		7	1145	6.1	0.90	(0.31, 2.57)	0.842	0.98	(0.34, 2.83)	0.964
CHF	22	976	22.5		16	1144	14.0	0.63	(0.33, 1.21)	0.164	0.48	(0.25, 0.96)*	0.037
Mortality	123	1009	122.0		89	1168	76.2	0.64	(0.48, 0.84)**	0.001	0.62	(0.47, 0.81)***	<0.001

PY: person-year; IR: incidence rate per 100 person-years; cHR: crude hazard ratio; aHR: adjusted hazard ratio; CI: confidence interval; MACE: major adverse cardiovascular event; ICH: intracerebral hemorrhage; CHF: congestive heart failure; LPS: lumboperitoneal shunt; VPS: ventriculoperitoneal shunt; ^ꝉ^: adjusted by sex, age, and all comorbidities; **: *p* < 0.01; ***: *p* < 0.001.

**Table 4:** MACE and mortality risk stratified by different follow-up times.

	Patients without shunting				Patients with VPS			Compare to patients without shunt							Patients with LPS			Compare to patients without shunt					
Outcome	N	PY	IR		N	PY	IR	cHR	(95% CI)	p-value	aHR^ꝉ^	(95% CI)	p-value		N	PY	IR	cHR	(95% CI)	p-value	aHR^ꝉ^	(95% CI)	p-value
MACE	28	715	39.2		61	868	70.3	1.84	(1.18, 2.89)**	0.008	1.83	(1.16, 2.90)**	0.010		57	1004	56.8	1.49	(0.95, 2.36)	0.084	1.41	(0.89, 2.24)	0.147
<1	9	232	38.8		33	243	135.9	3.50	(1.68, 7.32)***	<0.001	4.21	(1.96, 9.06)***	<0.001		22	256	85.9	2.22	(1.02, 4.83)*	0.044	2.10	(0.96, 4.58)	0.064
1-3	13	285	45.6		18	360	50.0	1.44	(0.68, 3.03)	0.336	1.23	(0.57, 2.66)	0.596		22	416	52.9	1.82	(0.90, 3.68)	0.093	1.83	(0.88, 3.80)	0.106
>3	6	198	30.3		10	265	37.7	1.11	(0.40, 3.12)	0.841	1.06	(0.34, 3.32)	0.914		13	332	39.2	1.22	(0.46, 3.26)	0.685	1.01	(0.35, 2.95)	0.988
Mortality	132	759	173.9		123	1009	121.9	0.74	(0.58, 0.95)*	0.018	0.74	(0.57, 0.95)*	0.020		89	1168	76.2	0.48	(0.37, 0.63)***	<0.001	0.45	(0.34, 0.59)***	<0.001
<1	73	235	311.3		41	258	159.0	0.52	(0.35, 0.76)***	<0.001	0.52	(0.35, 0.76)***	<0.001		26	268	97.1	0.32	(0.20, 0.50)***	<0.001	0.30	(0.19, 0.47)***	<0.001
1-3	34	302	112.4		49	410	119.6	1.62	(1.03, 2.53)*	0.036	1.75	(1.08, 2.83)*	0.022		31	469	66.1	1.25	(0.76, 2.05)	0.382	1.25	(0.75, 2.07)	0.396
>3	25	222.	112.6		33	342	96.6	0.87	(0.51, 1.47)	0.595	0.96	(0.55, 1.67)	0.873		32	431	74.2	0.71	(0.42, 1.21)	0.211	0.78	(0.45, 1.35)	0.376

PY: person-year; IR: incidence rate per 100 person-years; cHR: crude hazard ratio; aHR: adjusted hazard ratio; CI: confidence interval; MACE: major adverse cardiovascular event; LPS: lumboperitoneal shunt; VPS: ventriculoperitoneal shunt; ^ꝉ^: adjusted by sex, age, and all comorbidities; **: *p* < 0.01; ***: *p* < 0.001.

**Table 5:** Follow-up times of different outcomes.

	Without shunting			With VPS			With LPS	
	N=282			N=281			N=282	
Follow-up time, (year)	Mean	(SD)		Mean	(SD)		Mean	(SD)
MACE	3.32	(2.60)		3.09	(2.48)		3.56	(2.70)
Ischemic stroke	3.54	(2.61)		3.35	(2.57)		3.73	(2.64)
Hemorrhagic stroke	3.64	(2.71)		3.34	(2.57)		3.94	(2.81)
ICH	3.80	(2.75)		3.54	(2.60)		4.06	(2.88)
SAH	3.84	(2.73)		3.58	(2.65)		4.09	(2.79)
AMI	3.85	(2.78)		3.58	(2.65)		4.13	(2.89)
CHF	3.77	(2.78)		3.47	(2.61)		4.06	(2.92)
Mortality	3.87	(2.78)		3.59	(2.65)		4.14	(2.89)

MACE: major adverse cardiovascular event; ICH: intracerebral hemorrhage; SAH: subarachnoid hemorrhage; CHF: congestive heart failure; LPS: lumboperitoneal shunt; VPS: ventriculoperitoneal shunt; SD: standard deviation.
